# Clock-Enhancing Small Molecules and Potential Applications in Chronic Diseases and Aging

**DOI:** 10.3389/fneur.2017.00100

**Published:** 2017-03-15

**Authors:** Gabrielle F. Gloston, Seung-Hee Yoo, Zheng (Jake) Chen

**Affiliations:** ^1^Department of Biochemistry and Molecular Biology, The University of Texas Health Science Center at Houston, Houston, TX, USA

**Keywords:** circadian clock, small molecules, amplitude, metabolic disease, mood disorder, aging

## Abstract

Normal physiological functions require a robust biological timer called the circadian clock. When clocks are dysregulated, misaligned, or dampened, pathological consequences ensue, leading to chronic diseases and accelerated aging. An emerging research area is the development of clock-targeting compounds that may serve as drug candidates to correct dysregulated rhythms and hence mitigate disease symptoms and age-related decline. In this review, we first present a concise view of the circadian oscillator, physiological networks, and regulatory mechanisms of circadian amplitude. Given a close association of circadian amplitude dampening and disease progression, clock-enhancing small molecules (CEMs) are of particular interest as candidate chronotherapeutics. A recent proof-of-principle study illustrated that the natural polymethoxylated flavonoid nobiletin directly targets the circadian oscillator and elicits robust metabolic improvements in mice. We describe mood disorders and aging as potential therapeutic targets of CEMs. Future studies of CEMs will shed important insight into the regulation and disease relevance of circadian clocks.

## Introduction

The circadian clock is an intrinsic biological timing device operative in evolutionarily divergent species, ranging from microorganisms to human (1, 2). The clock drives daily oscillations of important molecular and physiological processes to anticipate and respond to the changing environment imposed by the rotation of the Earth. Consistent with its adaptive function, normal clock functions are required for organisms to survive and thrive. Coculture of cyanobacteria with varying period lengths demonstrated competitive growth advantage when inherent periodicity aligned with external light/dark rhythms (3), in accordance with findings from plant experiments (4). Likewise, circadian patterns of foraging and predator avoidance are well documented for animals in their natural habitats. For example, chipmunks whose central pacemaker, the hypothalamic suprachiasmatic nuclei (SCN), had been surgically removed suffered significantly higher mortality rate in the wild than those with fully functional clocks (5). The clock has also been postulated to protect early eukaryotes from irradiation during the day (6, 7). Despite the lack of acute lethality from genetic disruption of clock genes in laboratory animals, there exists a strong correlation, and in some cases causative relationship, between malfunctioning clocks and chronic diseases as well as aging (8, 9).

As we extend the list of clock-associated pathologies and probe for greater mechanistic understanding, the outstanding question remains whether and how to target the clock to combat disease and physiological decline (10–12). Except in the case of jet-lag, targeting the clock for health benefits will likely entail chronic intervention and gradual and systemic improvement of phenotypes and symptoms. Here, we highlight clock-associated metabolic disease, mood disorder, and aging as clock-associated processes characterized by dampened amplitude of circadian oscillation (13). Small-molecule enhancers of the circadian clock may strengthen the clock and clock-driven gene expression and physiology, retarding pathological deterioration. While this review will mainly focus on circadian amplitude enhancement, clock modulators capable of circadian phase and/or period modulation can show clinical utility in diseases states that are accompanied by circadian phase misalignment or abnormal periodicity (10, 14).

## Mammalian Circadian Clock

**Figure 1:**
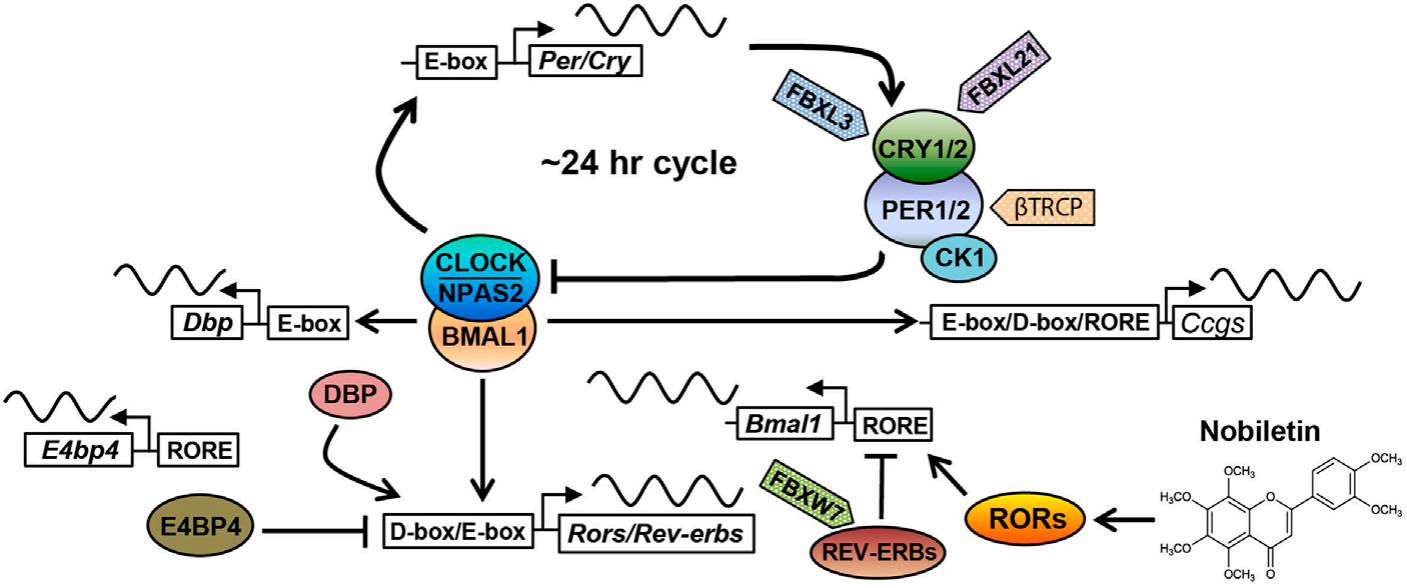
**The core circadian oscillator and regulatory molecules**. The circadian clock oscillator is comprised of a network of transcriptional–translational feedback loops including the core loop (BMAL1/CLOCK/NPAS2 and PERs/CRYs), the stabilization loop (BMAL1/CLOCK, REV-ERBs, and RORs), and the auxiliary loop (DBP, E4BP4, REV-ERBs, and RORs). Various protein regulators (F-box-containing E3 ligases are shown as examples) and small-molecule modulators (nobiletin is shown) have been identified to target core clock components, regulating circadian periodicity and amplitude. See the main text for details.

In the canonical mammalian clock, the molecular oscillator is the functional unit present in every cell of the body (15, 16). Comprised of interlocked feedback loops (Figure 1), molecular oscillators in individual tissues coordinate to govern highly tissue-specific expression programs of clock-controlled genes (CCGs). While 43% of genes have been shown to oscillate in at least one tissue in mice (17), indicating prevalent circadian gene regulation, the overlap of CCGs between tissues was found to be approximately 10% (18). At the system level, various tissue clocks are orchestrated by the SCN master pacemaker, a pair of neuron clusters bilaterally located in the anterior of the hypothalamus (19). The SCN displays tight coupling among its neurons (20) and functions to respond to photic signals to synchronize tissue and cellular clocks throughout the body *via* neural and hormonal signals.

The molecular oscillator is composed of intersecting negative feedback loops to drive ~24-h gene expression rhythms (1). In the core loop, the positive arm consists of three bHLH-PAS transcription factors, including paralogous CLOCK/NPAS2 and their heterodimeric partner BMAL1 (Figure 1). CLOCK or NPAS2 each interacts with BMAL1 through the PAS and bHLH domains. After dimerization, CLOCK/BMAL1 and NPAS2/BMAL1 activate expression of *Period* (*Per*) and *Cryptochrome* (*Cry*) genes *via* E-box promoter elements. PER and CRY proteins themselves heterodimerize and translocate into the nucleus to inhibit transcriptional activities of CLOCK/BMAL1 and hence their own transcription. CRYs belong to the photosensing photolyase protein family that functions in DNA damage repair in bacteria and in circadian photic entrainment in flies (21). However, the mammalian CRY proteins appear to have lost the photosensing ability yet acquired function as the major transcriptional repressor in the circadian core loop. Crystal structure studies showed that CLOCK and BMAL1 interact *via* bHLH and two PAS domains in an asymmetrical fashion, characterized by a β-sheet/α-helix interaction involving respective BMAL1 and CLOCK PAS-B domains (22). On the β-sheet surface of CLOCK PAS-B, mutagenesis screen identified several residues whose mutations attenuated CRY inhibition of CLOCK/BMAL1 transactivation, suggesting CLOCK/CRY interactions. Several crystal structures of CRY proteins have been reported. The FAD-binding domain of CRY proteins appears to be a key nodal point recognized by both a CRY-stabilizing small molecule (23) or an CRY-degrading E3 ligase (24), and that PER binding to CRY precludes access for the E3 ligase FBXL3 (25), thus stabilizing CRY. Future structural studies of core clock complex formation on promoter DNA will advance our understanding of circadian oscillator function.

Several other feedback loops have been shown to stabilize and/or modulate the core feedback loop (Figure 1). In the principal stabilization loop, CLOCK/BMAL1 and NPAS2/BMAL1 activate highly cyclic expression of genes encoding the nuclear hormone receptors REV-ERBα/β (26). REV-ERBs and their antagonistic receptors RORα/β/γ compete for binding to shared consensus elements (RORE and RevDR2) on the promoter of *Bmal1*/*Npas2* and other target genes throughout the genome to promote robust oscillatory gene expression (27–29). In another auxiliary loop (30), CLOCK/BMAL1 activates expression of genes encoding the PAR-bZip transcription factor DBP (D-box binding protein), which in turn drives *Ror* gene expression *via* their D-box promoter elements. In addition to *Bmal1*, REV-ERBs and RORs also govern the expression of the *Nfil3* gene, which encodes a transcriptional suppressor (also known as E4BP4) that binds to the D-box to antagonize DBP transcriptional activity. Apart from these transcriptional feedback loops, other feedback mechanisms are also important, including a post-translational loop involving the NAD+-dependent sirtuin (SIRT) 1 deacetylase (31). CLOCK/BMAL1 activates the *Nampt* gene, which encodes the rate-limiting enzyme for NAD+ biosynthesis. The NAD+ level directly correlates with SIRT1 activity, which directly target core clock proteins including BMAL1 and PER2 (32, 33).

Degradation of core clock components has emerged as a key regulatory mode for circadian functions. Casein kinase 1 has been shown to phosphorylate PERs, thereby facilitating their proteasomal degradation by the F-box proteins β–TRCP1/2 (34). Likewise, the AMPK kinase phosphorylates CRYs to promote CRY degradation (35), mainly mediated by the F-box protein FBXL3 (36–38). FBXL21, a close homolog of FBXL3, was found to antagonize FBXL3 to decelerate CRY degradation in the nucleus, on the other hand, also accelerate CRY turnover in the cytoplasm (39, 40). Mice harboring hypomorphic mutations in *Fbxl3* and *Fbxl21* showed opposite effects on circadian period length, highlighting an important circadian function for ubiquitin-mediated proteasomal degradation. Autophagy is another major protein degradation mechanism, involving lysosomal degradation of protein cargo delivered *via* autophagosome (41). It was recently found that BMAL1 undergoes dual degradation by proteasome- and autophagosome-dependent pathways, and attenuation of both in *Clock*Δ*19/*+ heterozygous mice improves glucose homeostasis (42). Overall, the circadian clock system is regulated by an exceedingly complex array of molecular mechanisms encompassing all levels of gene expression, together ensuring temporal precision (~24 h) and oscillatory robustness (see below).

## Circadian Amplitude Regulation

Amplitude denotes the robustness of circadian oscillation, measured by the difference between peak and trough of the circadian cycle. Whereas dampened circadian amplitude has been shown to closely correlate with chronic diseases and aging (10, 12, 43), the molecular and physiological mechanisms underlying circadian amplitude regulation are not well understood. Within the core oscillator, multiple lines of evidence indicated the importance of balancing positive vs. negative activities. For example, in mouse MEF cells, CLOCK/BMAL1 (positive factors) are in higher abundance than PER/CRY (the negative arm); as a result, overexpressing PER and CRY, but not CLOCK or BMAL1, strongly enhanced circadian amplitude (44). Such functional balance is further illustrated by the antagonistic transcriptional function of REV-ERBs and RORs in the secondary loop. Whereas ROR levels cycle only weakly, REV-ERB mRNA and protein levels are highly oscillatory. By directly competing for binding to promoter elements, they together govern a significant fraction of genome-wide circadian gene expression (29, 45). The clock is inherently a self-limiting, rhythmic machinery, namely, a limit cycle. Maintaining the “Yin–Yang” balance may lead to sustained oscillation, whereas brute force beyond a homeostatic range will dampen the overall amplitude of the following cycles. In other studies, CLOCK overexpression was found to enhance amplitude (46, 47), yet it remains unclear whether the primary mechanism involved is simply the greater level and activity of the positive transcription factor or an optimized functional balance.

More recent studies have provided insight into the functional complexity and dexterity of core clock components in amplitude regulation. In one study, REV-ERBα was found to be phosphorylated by cyclin-dependent kinase 1 (CDK1) at T275, a site not conserved in REV-ERBβ (48). Phosphorylated REV-ERBα was subsequently recognized by the F-box protein FBXW7 for proteasome degradation. Knockdown of CDK1 or FBXW7 reduced the amplitude of a circadian reporter in a dose-dependent manner, suggesting this REV-ERBα degradation pathway plays an important role in circadian amplitude. Another study described a “facilitated recruitment” mechanism where REV-ERBs are recruited to open chromatin following a rate-limiting step mediated by ROR/BMAL1 and transcription cofactors SRC-2/PBAF (49). It was posited that recruitment of the REV-ERB repressors by the activators ROR/BMAL1 ensures efficient and timely transcriptional shutdown, resulting in robust amplitude in target gene expression.

At intercellular and physiological/behavioral levels, oscillator coupling is of paramount importance to maintaining robust oscillation (50). The SCN rhythm is known to be exceptionally refractory to genetic perturbation compared with peripheral cells due to the tight coupling between SCN neurons (20). For example, several clock genes, including *Per1* and *Cry1*, are required for sustained PER2:LUC reporter rhythms in dissociated fibroblast cells and SCN neurons. At the tissue level, whereas lung explants remained arrhythmic, SCN slices showed robust cycling of the PER2:LUC reporter. In accordance, *Per1*-null mice displayed clear rhythmic locomotor behavior, albeit with a short period length (51). These studies together indicate that intercellular synchronization between SCN neurons, likely involving vasoactive intestinal polypeptide (VIP) (48), strengthens system amplitude. Such coupling-induced rhythm stabilization can also be observed in peripheral cells, where single-cell reporter rhythms were less robust or stable compared with those in tissue slices (16, 52). Besides genetic perturbation, intercellular coupling can also confer protection against pharmacological disturbance and stochastic noise (53). Reciprocally, intercellular coupling can also facilitate noise-generated stochastic rhythm. While dispersed SCN neurons from *Bmal1−/−* mice showed no circadian rhythmicity, *Bmal1−/−* SCN slices displayed shorter and highly variable circadian rhythms (54). Such unstable rhythms were shown to be abolished by tetrodotoxin-induced uncoupling in the SCN slices, further indicating that intercellular coupling augments rhythmic stability and robustness.

## Clock-Enhancing Small Molecules (CEMs) and Efficacies in Metabolic Disease Models

More than half of top-selling drugs act on protein targets encoded by cyclically expressed genes (17), and xenobiotic metabolism is subjected to circadian regulation (55). These findings indicate a close circadian regulation of pharmacodynamics and pharmacokinetics (56–58). On the other hand, rather than aligning the timing of chronotherapy with intrinsic rhythms, a distinct strategy is to manipulate the clock or clock components to alleviate clock-regulated disease symptoms (10–12, 14). Behavioral or dietary manipulations have been shown to modulate circadian rhythms, such as light exposure (59–61), exercise (62) as well as feeding/fasting regimens (63). For example, a series of studies have shown that time-restricted feeding (TRF) can improve sleep and metabolic homeostasis and delay cardiac aging in *Drosophila* (13, 64) At the molecular level, TRF activates genes involved in circadian rhythms and mitochondrial electron transport chain complexes. Similarly, timed caloric restriction (CR) led to highly consolidated food intake, which enhanced the expression and amplitude of core clock genes and improved lipid homeostasis, eventually contributing to life span extension (63, 65). Finally, bright light and melatonin, both major circadian synchronizers that strengthen rhythms, have been shown to improve cognition and mood in the elderly (66). These studies exemplify the beneficial effects of enhancing the molecular and physiological rhythms on physiology and behavior.

Various chemical compounds capable of manipulating clocks have been discovered *via* either unbiased phenotypic screens or targeted approaches focusing on particular clock components (67–72). As described above, the clock is a self-limiting machine with a myriad of check-and-balance mechanisms governing its periodicity and robustness. Excessive functional manipulation, either stimulatory or inhibitory, of a specific clock protein may compromise the inherent balance within the clock, eventually diminishing or even abrogating the intended effects. Therefore, when searching for small molecules capable of enhancing circadian robustness, it is important to evaluate the sustained effects on reporter rhythms rather than assaying only the molecular function of individual clock components. Below, we describe our recent efforts to utilize phenotypic screening to identify chemical modifiers that enhance circadian amplitude.

In two separate screens using cell-based phenotypic assays, we reported a group of clock amplitude-enhancing small molecules dubbed CEMs. The first screen of 200,000, largely synthetic, compounds identified 4 CEMs that potentiated cellular and tissue reporter rhythms in both WT and *Clock*Δ*19/*+ heterozygous mutant backgrounds (73). In contrast to *Clock*Δ*19/*+ heterozygous cells that displayed attenuated but sustained circadian rhythms, *Clock*Δ*19/*Δ*19* homozygous or *Bmal1*-null cells where the oscillators are essentially broken were refractory to CEM (14). CEM3, a benzimidazole compound, was uniquely able to further potentiate the robust reporter rhythms of the SCN pacemaker. In a second, smaller screen, a natural flavonoid compound called nobiletin (NOB) was identified as a novel CEM, along with its close analog tangeretin (74). NOB showed strong enhancing activities in circadian reporter cells, with an EC50 in the low micromolar range. NOB is a major polymethoxylated flavone found in citrus peels and exhibits a favorable pharmacokinetic profile devoid of significant toxicity (75). Previous studies have reported diverse biological activities against metabolic syndrome, oxidative stress, inflammation, and cancer (76–80); however, its molecular mechanism of action and direct protein targets were unknown.

A potential metabolic efficacy of NOB is intriguing and provides a focal point of connecting circadian manipulation and metabolic fitness. Previous research has established a regulatory role of the circadian clock in metabolic homeostasis (31). For example, the *Clock*Δ*19/*Δ*19* mutant mice showed a broad array of metabolic dysfunctions, including blunted feeding rhythms, hyperphagia, exaggerated obesity risk under high-fat diet (HFD) feeding or at older ages, elevated blood glucose levels and hypoinsulinemia (81). Reciprocally, metabolism and/or nutrition also modulate our internal clocks (82, 83). For example, under *ad libitum* HFD feeding, mice showed a slight increase in the free-running period length (~23.8 h) compared with regular chow-fed animals (~23.6 h), and importantly a marked decrease in amplitude of circadian rhythms, including both clock gene oscillation in the periphery and feeding rhythms (82, 84). Both examples showed a correlation of circadian amplitude reduction and metabolic dysfunction, consistent with human studies where blunted insulin secretion rhythm associates with increased risk for diabetes (85).

We therefore examined the efficacy of NOB in two mouse metabolic disease models, namely the HFD-induced obese mice and *db/db* diabetic mice. Metabolic characterization illustrated that NOB effectively mitigated body weight gain without altering food intake, stimulated energy expenditure (EE) and circadian activity, enhanced glucose and insulin tolerance, and diminished lipid content in circulation and in liver (74). The alleviated liver steatosis phenotype was accompanied by restored oscillation of core clock components in mouse liver. In addition to energy homeostasis, NOB was also found to reduce serum ammonia levels in different diets and appeared to enhance urea cycle gene expression and function under HFD feeding (86). *Clock*Δ*19/*Δ*19* homozygous mutant mice showed no or much diminished response to NOB, indicating clock requirement for NOB effects. Microarray analysis using mouse liver showed extensive remodeling of energy metabolic pathways including lipid metabolism and mitochondrial respiration. Together, these findings support the notion that clock enhancement by NOB contributes to metabolic improvement (87).

Importantly, NOB was found to directly activate ROR receptors *via* filter binding and functional studies including mammalian one-hybrid assays (74). This key finding highlights the role of RORs in circadian amplitude regulation and also sheds important insight on the functional complexity of NOB and ROR. First, despite the robust affinity of NOB–ROR interaction, the activation of ROR target genes, including core clock genes (e.g., *Bmal1*) and downstream output genes, was generally moderate (74). This observation is consistent with the limit cycle nature of the clock where the balance between positive and negative limbs is paramount to the overall amplitude. Second, a large number of ROR inverse agonists and REV-ERB agonists have been identified (71, 88). Despite opposite molecular functions relative to NOB as an ROR agonist, several of these compounds have been shown to improve energy metabolism in metabolic disease models (89, 90). This apparent paradox illustrates a potential functional dexterity of ROR (and also REV-ERB). It is possible that specific ligands, either agonists or antagonists, of ROR/REV-ERB can promote metabolic health, likely *via* distinct compound-specific mechanisms. A recent study (91) showed that three antagonists of RORγt employed divergent molecular mechanisms to affect its promoter binding and target gene expression and exhibited different degrees of mimicry with genetic RORγt disruption. These studies highlight the importance of in-depth mechanistic understanding of CEMs in circadian rhythms and downstream physiology.

## Mood Disorders and Aging as Potential Pathophysiological Targets of CEMs

Below we highlight two potential targets of CEMs, namely mood disorders and aging, where accumulating evidence indicates a strong correlation between pathophysiology and clock amplitude decline.

### Mood Disorders

Mood disorders and circadian dysfunction are closely associated. Various manifestations of major mood disorders such as major depressive disorder, bipolar disorder, and seasonal affective disorder (BPD and SAD, respectively) exhibit diurnal rhythms, with the most severe symptoms typically occurring in the morning or around sunset (92, 93). In an early study comparing depressed, recovered, and healthy subjects, the depressed group exhibited blunted circadian rhythms, with a significant correlation to scores on depression severity (94). Recovered participants following 3 weeks of antidepressant treatment showed restored circadian amplitude, suggesting that depression is closely linked to circadian rhythmicity. In SAD patients suffering from depression during winter months with shorter daytime (95), circadian rhythms in feeding, sleep, body temperature, cortisol, and melatonin release, neurotransmitter (serotonin, norepinephrine, and dopamine) have been shown to be disturbed or dampened (96, 97). Another mood disorder is Sundowning syndrome, also referred to as “nocturnal delirium” (93). Sundowning syndrome is characterized by a worsening of behavior (i.e., aggression, restlessness, delirium, and agitation) in the late afternoon or early evening, particularly in the elderly population suffering from dementia. Clinical and preclinical data suggest that disturbances in sleep, environmental entrainment cues, and the SCN pacemaker all contribute to Sundowning syndrome (93). Specifically, sleep disruptions including impaired NREM sleep consolidation, sleep fragmentation, daytime sleeping, and reduced sleep efficiency are common among both the elderly and demented (98), and circadian amplitude disturbances manifested as sleep disruptions listed above can contribute to mood imbalance (99).

Mouse studies have begun to supply evidence for a possible causal relationship between clock function and mood. For example, behavioral assays using the *Clock*Δ*19/*Δ*19* mice revealed manic-like behaviors similar to human bipolar mania (100), including hyperactivity, decreased sleep, hyperhedonia, and an increased preference for cocaine use. Disrupted circadian rhythms are also commonly found in human mania (94). More recently, the subcapsular cell hyperplasia associated with adrenal tissue remodeling was reported to enhance circadian amplitude of glucocorticoid rhythm, but not the total glucocorticoid levels (101). Interestingly, the enhanced stress hormone rhythm promotes anxiolytic function. It was postulated that the high-amplitude oscillation of the anxiogenic glucocorticoid, the descending phase in particular, endows a robust anxiolytic response to regulate mood balance.

Consistent with a close relationship between clock disruption and mood disorders, various treatment options are known to manipulate or enhance circadian and/or sleep cycles. Among the environmental therapies are bright light therapy, social rhythm therapy (SRT), and sleep deprivation. Bright light therapy is the treatment of choice for SAD and has also been applied to depression, bipolar disorder, and sleep–wake cycle disturbances (102). Bright light in the morning serves to advance the circadian phase to correct the phase delays commonly seen in SAD patients and may also function as a strong photic zeitgeber to improve daily rhythms. Likewise, SRT (103) entails social zeitgebers such as routine daily tasks to restore stability of biological rhythms in depression patients. Finally, a total sleep deprivation paradigm has also been developed to temporarily alleviate SAD symptoms. Its biological basis is not well understood, although it has been shown to impact neurotransmitter function and rapidly reset behavioral and circadian rhythms (104). Therefore, behavioral and environmental cues employed in these therapies reset and potentiate circadian rhythms, mainly at the behavioral levels, to counter the debilitating depressive tendency.

Various pharmacological agents have been used in mood disorders, including antidepressants, antimanic or mood-stabilizing drugs, and antipsychotics (Table 1). Lithium is a mood-stabilizing drug that has been used to treat bipolar disorder for more than 50 years. In addition to its mood-stabilizing effects, lithium has been reported to lengthen the free-running circadian period in mammals including hamsters and mice (105, 106). A potentially important target of lithium is GSK-3β (107), a kinase broadly acting in various signaling pathways. GSK-3β was previously shown to phosphorylate and stabilize REV-ERBα, and lithium treatment accelerated proteasomal degradation of REV-ERBα (108). More recently, lithium was found to activate *Per2* gene expression and enhance the circadian reporter amplitude in both SCN and periphery (106). Another pharmacological treatment that affects the circadian system is valproic acid or valproate. Valproate is traditionally an anti-epileptic drug but has been repurposed as a mood-stabilizing drug. Valproate has been shown to alter circadian period (109) and acute valproate treatment of PER2:LUC bioluminescence experiments in skin fibroblasts yielded amplitude enhancement and induced phase-shifts, depending on the relative level of PER2:LUC protein expression (110). Previous mouse studies have also suggested antidepressive functions of NOB (111, 112) (Table 2). For example, NOB was found to improve mouse performance in forced swimming test and tail suspension tests, while pretreatments with drugs targeting monoaminergic systems disrupted the NOB effects (112). It will be interesting for future studies to investigate a role of circadian clocks in these NOB efficacies.

**Table 1:** **Pharmacological treatments for mood disorders targeting the circadian system**.

Drug name	Therapeutic effect	Circadian target(s)	Circadian-related effect(s)	Reference
Lithium	Mood stabilizer	GSK-3β	Lengthened circadian period; enhanced PER2 protein expression; and oscillatory amplitude	(105, 106)
Valproate	Mood stabilizer	Dopamine-mediated, possibly PER2	Shortened circadian period of behavioral rhythms in DAT-KD mice and rhythms in suprachiasmatic nuclei explants from PER2:LUC mice	(109)
Quetiapine	Mood stabilizer; adjunctive antidepressant; antipsychotic	Per1/2, Bmal1	Enhanced Per1/2 mRNA at different ZTs in the mouse amygdala	(113)
Carbamazepine	Mood-stabilizer	Undetermined	Shortened length of locomotor activity; stabilized running activity	(114)
Fluoxetine	Antidepressant	Per2/3, Cry2, GSK-3β	Altered circadian period; enhanced hippocampal clock gene expression; altered phase re-entrainment	(115–117)
Agomelatine	Antidepressant	MT1/2 receptors	Accelerated resynchronization of circadian rhythms; improved rest–activity cycle more than common antidepressant; entrained circadian rhythms; induced phase-shifts	(118–123)
Ramelteon	Antidepressant	MT1/2 receptors	Phase advance	(124)
Tasimelteon	Antidepressant	MT1/2 receptors	Phase advance/delay	(125)

**Table 2:** **Antidepressive and neuroprotective roles of nobiletin**.

Species	Treatment duration	Effect	Cellular effects	Reference
Mouse (despair model *via* FST and TST)	60 min prior to assay	Antidepressant	Monoamine upregulation	(112)
Mouse	11 days	Antidepressant; improved memory impairment	Activated ERK/MAP kinase-dependent signaling and increased CREB phosphorylation	(111)
Mouse AD (APP-SL 7-5 Tg mice)	4 months	Reduced Aβ plaque pathology; improved memory impairment	ERK phosphorylation; enhanced neprilysin activity	(126)
Mouse AD (3XTg-AD)	3 months	Improved cognitive impairment	Reduced soluble Aβ levels, reduced ROS levels in the hippocampus of WT and 3XTg-AD mice	(127)
Mouse (senescence-accelerated mouse prone 8, SAMP8)	2 months	Improved recognition and context-dependent fear memory	Restored decrease in GSH/GSSG ratio, increased antioxidant (GPx) enzyme activity, reversed tau phosphorylation at Ser202 and Thr231	(128)
MPTP-treated model mice	14 days	Improved motor and cognitive deficits	Increased levels of CaMKII autophosphorylation and phosphorylation of DARPP-32 in the striatum and hippocampus; restored CaMKII- and cAMP kinase-dependent TH phosphorylation; enhanced dopamine release in striatum and hippocampus	(129)

*Future studies are required to delineate the role of circadian clock in these efficacies*.

*FST, forced swim test; TST, tail suspension test*.

### Aging

Gradual decline in metabolic, physiological, and behavioral functions with age leads to increasing risk of chronic disease and mortality (130). One physiological basis for such system-wide deterioration is age-related circadian attenuation (13, 43). Various clock-regulated physiological and behavioral processes are known to display reduced amplitude with age (43, 61, 131). For example, aging correlates with impaired rhythms in SCN firing rate, hormone secretion (e.g., cortisol and melatonin), and body temperature (132). Sleep fragmentation, characterized by multiple short periods of sleep episodes throughout the normal sleep phase and also sleep during the normal active phase, indicates amplitude dampening of the sleep/wake cycle and constitutes a well-documented characteristic of aging and various age-related diseases including Alzheimer’s disease (133). At the molecular level, there is also broad dysregulation of clock gene expression (61, 134, 135). Whereas peripheral clocks appear to suffer amplitude dampening (136, 137), the central clock neurons maintain robust molecular oscillation (135, 137). It is possible that cellular coupling and/or output pathways are compromised during aging, leading to systemic decline. In accordance, old age in both humans and mice is associated with delayed adaptation to phase shift cues (138, 139), suggesting that aging compromises circadian synchronization and weakens entraining response. Genetic studies have also provided evidence linking the clock and aging. The *Bmal1*-null mutant mice, exhibiting arrhythmic clock gene expression and defective clock-controlled physiological processes such as metabolism and activity (140, 141), suffered premature aging phenotypes such as sarcopenia, cataracts, and early mortality (142, 143). On the other hand, the αMUPA transgenic mice, as a long-living mouse model, displayed 24-h circadian periodicity regardless of age (144). These mice maintained robust behavioral and physiological rhythms, and core clock gene expression showed enhanced amplitude. Collectively, the evidence indicates that circadian robustness, involving both clock gene oscillation and systemic synchronization (145), may confer beneficial effects on life span and health span.

An established circadian output marker is melatonin (146), a sleep-regulating hormone in humans whose synthesis pathway is governed by the clock (147). Aging dampens the circadian peak (and amplitude) and daily total secretion of melatonin (148–150), contributing to lower sleep quality including decreased rapid eye movement, slow wave sleep, and increased stage 2 non-REM sleep in the elderly (151, 152).

Aging is associated with prevalent metabolic deterioration (130). For example, total EE declines during aging, as the elderly display diminished EE and gross energy intake (EI) compared with young adults (153). Such age-related energy imbalance, with EI > EE in the elderly and EI < EE in young adults, causes exaggerated body mass index during aging (154). Body temperature is a circadian output that shows a diurnal pattern with a dip during sleep (146, 155, 156). Thermogenesis plays a significant role in energy homeostasis, and age-related deterioration in energy homeostasis impairs circadian body temperature rhythm. For example, despite largely comparable basal body temperature, phase and amplitude of body temperature rhythm have been shown to significantly differ between the elderly and young- or middle-aged subjects (155, 157, 158). Liver and muscle play important roles in body temperature regulation, and attenuated skeletal muscle mass and mitochondrial function significantly contribute to dampened energy homeostasis and thermogenesis during aging (62, 157).

Caloric restriction universally prolongs life span (159). CR depletes white adipose tissue, especially the pro-inflammatory and diabetogenic visceral fat that accumulates over age (160). Timed CR leads to highly consolidated food intake within a few hours, enhancing the amplitude of circadian metabolic rhythms (63, 161) and core clock gene oscillation (65). CR involves several nutrient-sensing pathways including AMPK, AKT, and mTORC1, all of which have been reported to functionally interact with the clock (31, 42, 160, 161). In particular, the NAD+-dependent deacetylase SIRT proteins play important roles at the interface of energy homeostasis, clock, and aging (161, 162). Mammals express seven SIRT proteins (SIRT1–7), several of which have been implicated in circadian regulation of metabolism (32, 33, 163, 164). For example, SIRT1 directly deacetylates core clock components including BMAL1 and PER2, regulating their molecular function and CCG expression (32, 33). More recently, SIRT1 was found to interact with PGC-1α to control *Clock* and *Bmal1* gene expression in the SCN, consequently regulating CLOCK/BMAL1 target genes (165). Various SIRT1-activating small molecules (e.g., resveratrol) have been shown to extend life span (166); resveratrol, in particular, has been shown to modulate physiological and behavioral rhythms and clock gene expression (167–169).

## Future Directions and Concluding Remarks

Circadian amplitude regulation and pharmacological modifiers are exciting research topics with promising translational potential. The list of CEMs will likely continue to grow, either from phenotypic screening, as in the case of NOB, or from targeted ligand development (14). On the other hand, pharmacological agents shown to target or mimic clock-enhancing pathways such as CR, TRF, and exercise are a rich venue for discovery of additional clock-targeting agents (63, 130, 161, 170). For example, a growing number of small molecules or drugs have been shown to extend life span and health span, including those deliberately designed to mimic CR and other manipulations (170, 171). Future studies should characterize their circadian clock effects and delineate molecular mechanisms.

Besides metabolic diseases, mood disorders, and aging, other chronic diseases such as neurodegenerative diseases (172, 173) have also been shown to correlate with dampened circadian amplitude or clock dysregulation and may represent new venues for studies of clock modifiers. In addition to antidepressive effects, several studies have shown neurological efficacies of NOB using transgenic disease models (Table 2). For example, 11-day oral administration of NOB resulted in an overall memory improvement in olfactory-bulbectomized (OBX) mice based on the step-through passive-avoidance task and the Y-maze test (111). OBX mice share clinical features with both human neurodegenerative diseases and major depression (174). The depression-like phenotype is thought to derive from pathological or compensatory mechanisms within the cortical–hippocampal–amygdala circuit, which typically involve deterioration of spine density and/or synaptic strength changes (175). Future studies are required to determine the specific role of circadian clocks and RORs in disease models.

Significant gaps of knowledge remain regarding circadian amplitude regulation, especially the mechanisms employed by CEMs. At the intracellular level, questions of particular interest include gene expression regulation, such as cofactor recruitment, epigenetic mechanisms, and chromosome dynamics (1). At the intercellular and system levels, other coupling molecules in addition to VIP and the communication between peripheral and central clocks are outstanding questions (50). It is conceivable that CEMs execute distinct mechanistic schemes to restore a robust overall output under disease or aging conditions. Exemplified by the complex and divergent ROR mechanisms when bound by distinct ligands (74, 90, 91), a detailed mechanistic understanding is important to fully exploit the therapeutic potential of individual CEMs.

In conclusion, circadian clocks safeguard physiological health, and dysregulated and dampened clocks can serve as therapeutic targets to mitigate disease symptoms. Exciting functional and mechanistic studies await to develop CEMs as novel preventive and therapeutic agents.

## Author Contributions

GG, S-HY, and ZC contributed to manuscript preparation.

## Conflict of Interest Statement

The authors declare that the research was conducted in the absence of any commercial or financial relationships that could be construed as a potential conflict of interest.
